# Diagnosis, Classification and Management of Ankyloglossia Including Its Influence on Breastfeeding

**DOI:** 10.34763/devperiodmed.20192301.7985

**Published:** 2019-04-08

**Authors:** Dagna Brzęcka, Małgorzata Garbacz, Marcin Micał, Barbara Zych, Bogumił Lewandowski

**Affiliations:** 1 Department of Maxillofacial Surgery of the F. Chopin Clinical Voivodship Hospital in Rzeszow, Rzeszow Poland; 2 Medical Faculty of the University of Rzeszow, Rzeszow Poland; 3 NZOZ Primadent in Rzeszow, Rzeszow Poland

**Keywords:** ankyloglossia, breastfeeding, frenulotomy, ankyloglosja, karmienie piersią, frenulotomia

## Abstract

Ankyloglossia is defined as a congenital malformation that alters lingua/ mobility and function. ft is listed as one of the possib/e reasons behind problems with breastfeeding. Due to current WHO recommendations that encourage mothers to breastfeed exclusively up to **6** months of age, quick recognition of any obstacles in the suction mechanism and determining the possible reasons for problems should be a priority. A review of the literature was conducted concerning the diagnosis of ankyloglossia, possible methods of treatment and their efficacy in improving breastfeeding quality. The authors of the research cited claim that any surgica/ intervention should be performed only in cases of symptomatic ankylog/ossia interfering with sucking mechanisms. The most frequent surgical procedure performed in newborns with symptomatic ankyloglossia is frenulotomy. ft is a simple procedure with a low risk of complications. The literature gives a great number of studies confirming both the short and long-term efficacy of tongue-tie release in improving breastfeeding quality with emphasis on decreasing mothers' discomfort, nipple pain and trauma.

## Streszczenie

Ankyloglosja definiowana jest jako wrodzone zaburzenie rozwojowe ograniczające ruchomość i funkcję języka. Wymieniana jest jako jedna z możliwych przyczyn trudności w karmieniu piersią. Ze względu na aktualne wytyczne WHO, które zalecają wyłączne karmienie naturalne noworodka do **6**. miesiąca życia, kluczowe jest wczesne wykrycie ewentualnych zaburzeń ssania i ustalenie ich przyczyny. Dokonano przeglądu piśmiennictwa dotyczącego diagnostyki skróconego wędzidełka języka, metod leczenia i ich skuteczności w poprawie karmienia naturalnego. Autorzy prac podkreślają konieczność ograniczenia ewentualnej interwencji chirurgicznej tylko do przypadków objawowej ankylog/osji zaburzającej proces ssania. U noworodków najczęściej wykonywany jest zabieg prostego podcięcia wędzidełka frrenulotomii), który jest szybki i nie obarczony poważnymi powikłaniami. Literatura dostarcza wielu dowodów na skuteczność zabiegu frenulotomii w poprawie jakości karmienia piersią, a szczególnie podkreślany jest wpływ na zmniejszenie dyskomfortu matki podczas karmienia, bólu i urazów brodawki sutkowej.

## Introduction

A short lingual frenulum (ankyloglossia, tongue-tie) is defined as a congenital malformation that limits lingual mobility, thus leading to impaired tongue function [1,2]. Its prevalence in newborns is estimated in the literature at between 4.2% and 10.7% [3, 4, 5], with the male to female ratio of 3:1 [5, 6]. The discrepancies in tongue-tie frequency assessment are most probably caused by the lack of unambiguous and objective diagnostic criteria, as well as significant differences in study groups [2].

## Aim

The aim of this study was to review the literature regarding current recommendations for the diagnosis of ankyloglossia in newborns, its potential impact on breastfeeding, indications for surgical treatment and its efficacy.

## Material and methods

Areview of the literature from peer-reviewed journals was conducted by searching the PubMed database using the keywords: ’’ankyloglossia”, ’’tongue-tie” and ’’frenulotomy”. The 34 best-matching references were selected with emphasis on the most recent studies. Most of them were published between July 2012 and April 2018. Former publications cover topics that have not been raised in more recent studies.

## Results

During the developmental period, the tongue plays an important role in the proper growth of the oral cavity and the whole stomatoghnathic system. Many studies claim there is an influence of a short lingual frenulum on the development of malocclusions (especially class III disorders, maxillary hypoplasia, open bite, mandibular diastema and mouth-breathing) [7, 8, 9], as well as altered posture [8]. Moreover, a disfunction of the tongue that coexists with ankyloglossia may lead to speech defects as a result of incorrect articulation [10]. Lalakea and Messner state, however, that it is not a potential cause of delayed speech development or stuttering [1]. Nevertheless, preventive frenulotomy that aims to avoid the previously mentioned disorders is still a controversial issue. Undisturbed tongue function in the neonatal period is also essential in developing proper suction during breastfeeding. The literature widely describes the influence of ankyloglossia on difficulties in breastfeeding. According to Messner et al. [3] 25% of the newborns with a short lingual frenulum experience such problems. Ferrés-Amat et al. [11], who studied a group of Spanish infants with diagnosed natural feeding difficulties estimated the prevalence of ankyloglossia at 15%. A short lingual frenulum may impede proper nipple latch and seal during breastfeeding. Altered sucking mechanics leads to low milk transfer and its low supply, prolonged feedings, poor weight gain, tiredness and irritation of the child during breastfeeding, as well as nipple pain and trauma that is caused by the pressure of the newborn’s gingiva [2, 4, 5, 6]. Such problems may provoke the mother’s discouragement, thus leading to premature weaning and switching to a bottle [2]. On the other hand, those difficulties are not observed in bottle-fed newborns with ankyloglossia [5]. WHO recommends exclusive breastfeeding up to 6 months of age as the most beneficial for mother-infant dyads [12]. During breastfeeding peristaltic muscle movements are activated, which stimulates the proper development of the structures of the stomatognathic apparatus. During bottle-feeding such stimulation is limited [11]. Therefore, it is crucial to encourage mothers to breastfeed, to focus on the early diagnosis of any obstacles and their plausible reasons, including ankyloglossia, and to apply proper treatment as soon as possible.

Examination of the newborn’s oral cavity should include the assessment of tongue appearance, shape, position and function both in relaxation and when moving, frenulum elasticity, length of the free tongue as well as the size of lingual frenulum attachments to the tongue, the floor of the mouth and the inferior alveolar ridge. The attachment of the frenulum to the tongue should normally be approximately 1 cm posterior to the tongue’s tip [2]. One of the most common characteristics of ankyloglossia is a heart-shaped tongue tip when the tongue is lifted. Besides assessing tongue appearance, it is essential to properly examine the tongue’s function in the course of movement, while the newborn cries or sucks a finger. Thorough palpation of the tongue frenulum to check its reaction to lateral and posterior pressure is especially important in diagnosing ankyloglossia posterior (posterior tongue-tie), in which a short lingual frenulum is hidden under mucosa and anchors only the middle part of the tongue leaving a free tip, which significantly impairs tongue function and peristalsis [13]. In the study by Ghaheri et al. [14] 78% of newborns experiencing problems with breastfeeding were diagnosed with ankyloglossia posterior.

The lack of a universal, unequivocal and commonly accepted definition and objective diagnostic criteria of ankyloglossia is still an issue. There are many classifications for lingual frenulum assessment in newborns proposed in the literature. Kotlow [15] introduced a simple anatomical classification based on measuring the “free tongue” length (between the frenulum attachment to the tongue and the tongue’s tip) (tab. I).

**Table I:** Classification of ankyloglossia according to Kotlow (based on the "free tongue" length).

Normal, clinically acceptable range of "free tongue" >16mm	
Class 1: mild ankyloglossia	12-16 mm
Class II: moderate ankyloglossia	8-11 mm
Class III: severe ankyloglossia	3-7 mm
Class IV: complete ankyloglossia	<3 mm

The study by Walker et al. [16] demonstrated a correlation between the tip-frenulum length and difficulties in breastfeeding which manifested mainly as mothers’ nipple pain. The average “free tongue” length in this study, in a group of 100 generally healthy newborns was found to be 9.07mm. Coryllos’ classification [17] also includes types III and IV of ankyloglossia, which meet the diagnostic criteria of ankyloglossia posterior. Due to their uncharacteristic appearance, they may easily remain unrecognized on examination (tab. II).

**Table II:** Classification of ankyloglossia according to Coryllos.

Type 1	Fine and elastic frenulum; the tongue is anchored from the tip to the alveolar ridge and it is found to be heart-shaped
Type II	Fine and elastic frenulum; the tongue is anchored 2-4 mm from the tip near the alveolar ridge
Type III	Thick, fibrous non-elastic frenulum; the tongue is anchored from its middle to the floor of the mouth
Type IV	The frenulum cannot be seen but palpated; it has a fibrous and/or thick and shiny submucous anchoring from the base of the tongue to the floor of the month

However, there is no confirmed correlation between the type of frenulum according to Coryllos’ classification and the degree of breastfeeding problems [18]. The Hazelbaker questionnaire [19] (The Hazelbaker Assessment Tool for Lingual Frenulum Function – HATLFF), in turn, presented in the form of a point scale, includes both anatomical (5 items) and functional (7 items) criteria. Significant ankylogossia, which requires frenulotomy, is diagnosed with the appearance score <8, and the function score <11 (tab. III).

**Table III:** Diagnostics of ankyloglossia according to Hazelbaker (Hazelbaker, The Assessment Tool for Lingual Frenulum Function).

Function items	Appearance items
Lateralization	**Appearance of tongue when lifted**
2 – complete	2– round or square
1 – body of tongue but not tongue tip	1 – slight cleft in tip apparent
0–none	0– heart-shaped
Lift of tongue	
2–tip to mid-mouth	**Elasticity of frenulum**
1 – only edges to mid-mouth	2–very elastic
0 – tip stays at alveolar ridge or tip rises	1 – moderately elastic
to mid-mouth with jaw closure	0 – little or no elasticity
**Extension of tongue**	**Length of lingual frenulum when tongue lifted**
2–tip over lower lip	2–>1 cm
1 – tip over lower gum only	1–1 cm
0 – neither of the above or mid-tongue humps	0–<1 cm
**Spread of anerior tongue**	**Attachment of lingua frenulum to tongue**
2 – complete	2 – posterior to tip
1 – partial	1 – at tip
0– little or none	0– notched
**Cupping of tongue**	**Attachment of lingual frenulum to inferior alveolar ridge**
2 – entire edge, firm cup	2 – attached to floor of mouth or well below ridge
1 – side edges only, moderate cup	1 – attached just below the ridge
0 – poor or no cup	0– attached at ridge
**Peristalsis**	
2 – complete anterior to posterior (originates at tip)	
1 – partial (orignates posteriori to tip)	
0 – none or reverse peristalsis	
**Snap-back**	
2 – none	
1 – periodic	
0 – frequent or with each suck	
**Scoring**:	
14 – perfect score	
11 – acceptable if appearance item score is 10	
<11 – tongue function impaired (frenotomy should be considered)	
**Frenotomy is necessary if function score is <11 and appearance score is <8**	

Nevertheless, an unambiguous correlation between the Hazelbaker score and the degree of breastfeeding difficulties has not been confirmed. Moreover, it is found that problems with breastfeeding are not directly correlated with lingual frenulum appearance, and may occur in both

anterior and posterior ankyloglossia [6, 20]. According to Ricke et al. [5] 80% of the newborns with diagnosed ankyloglossia are able to effectively suck a breast without any surgical intervention, due to the tongue’s adaptability, despite the incorrect appearance of the lingual frenulum. In the study by Kumar et al. [21] half of the examined children with a short lingual frenulum were diagnosed with asymptomatic ankyloglosia that did not cause any obstacles in breastfeeding. Therefore, in order to adequately qualify for surgical intervention, it is necessary to include breastfeeding criteria. Surgical procedure itself should be performed only in those ankyloglossia cases in which breastfeeding difficulties occur [6]. FDRBI (Frenotomy Decision Rule for Breastfeeding Infants) proposed by Srinivasan et al. [22] may be a helpful tool. According to the questionnaire, only a short lingual frenulum that coexists with breastfeeding difficulties (persistent nipple pain and/or inadequate latch and/or poor weight gain <15 g per day) and improper tongue function (inability to protrude beyond the alveolar ridge, to lift the tongue to the roof of the mouth or to adequately cup the nipple-areolar complex) may be qualified for frenulotomy. Moreover, the LATCH index may also be applied for objective and quantitative assessment of breastfeeding quality, which includes 5 categories: L (latch), A (audible swallowing), T (type of nipple), C (comfort) and H (hold) and scores them from 0 to 2. Table IV [23] presents an accurate description of the index, together with proposed example questions that may be helpful during conversations with mothers that experience breastfeeding problems.

**Table IV:** LATCH scale.

	0	1	2	Example questions
L– latch	Too sleepy or reluctant No latch obtained	Repeated attempts Must hold nipple in mouth Must stimulate to suck	Grasps breast Tongue down and forward Lips flanged Rhythmic suckling	How did your baby grasp your breast? Did you baby suckle on his own or did you have to work with him?
A–audible swallow	None	A few with stimulation	Spontaneous	Did your you baby hear swallow? How frequently?
T– type of nipple	Inverted	Flat	Everted (after stimulation)	Do your nipples stand out or do they flatten easily?
C– comfort	Engorged Cracked, bleeding, large blisters or bruises Severe discomfort	Filling Small or bruises blisters Mother complains of pinching Mild/moderate discomfort	Soft Tender Intact nipples (no damage)	Are your nipples tender? Are your breasts becoming full and heavy?
H– hold	Full assist (Staff holds infant at breast)	Minimal assist (i.e. elevate head of bed, place pillows) Teach one side mother does other Staff help, mother takes over feeding	No assist from staff Mother able to position/ /hold infant	Did someone help you put your baby to breast? Do you need help with the next feeding?

Infant Breastfeeding Assessment Tool (IBFAT) is a similar scale, that consists of 4 questions for mothers describing their experience during breastfeeding, scored from 0 to 3 (tab. V) [16].

**Table V:** Infant Breastfeeding Assessment Tool (IBFAT).

	3	2	1	0
To get the baby to begin this feed, did you have to:	Just place the baby on the breast, as no effort was needed	Use mild stimulation, such as unbundling, patting, burping	Unbundle baby, sit baby back and forward, rub baby's body or limbs at the beginning and during the feed	Could not be aroused
Rooting (at touch of nipple to cheek, baby's head turns toward the nipple, the mouth opens, and baby attempts to fix mouth on the nipple) When the baby was placed at the breast he/she:	Rooted effectively at once.	Needed some coaxing, prompting/ /encouragement to root	Rooted poorly even with coaxing	Did not try to root
How long from placing baby he/she at the latch breast does on and start to feed well?	0–3 minutes	3–10 minutes	Over 10 minutes	Did latch not at all
Which of the following phrases best describes the baby's feeding pattern at this feed?	Sucked well on one or both breasts	Sucked fairly well (sucked off and on but needed some encouragement)	Sucked poorly, weak sucking, some sucking for short periods	Baby did not suck

The study by Altuntas et al. [24] confirms the reliability of both indexes (LATCH and IBFAT) in breastfeeding quality assessment.

The most frequent surgical procedure performed in newborns with symptomatic ankyloglossia is frenulotomy. The lingual frenulum is poorly innervated and vascularized, thus the procedure normally does not require local anesthesia [2]. Alternatively, a topical anesthetic may be applied. However, its effectiveness in pain control during tongue-tie release has been questioned in the literature [25]. The procedure itself involves cutting the lingual frenulum with surgical scissors after stabilizing the tongue with the fingers or spatula. The incision should begin at the free margin of the frenulum and proceed posteriorly adjacent to the tongue with care not to injure inferiorly based submandibular salivary ducts. The mild bleeding that may occur after the surgery can be controlled with gauze and light pressure, no suture is required. It is recommended to breastfeed immediately after the procedure [2], while during the next 7-10 days tongue mobilizing exercises that prevent reattachment of the frenulum are advised [26]. Nowadays diode laser is increasingly popular as an alternative in tongue-tie release [8, 14, 27]. Its application decreases the bleeding in the operating field, minimalizes swelling and post-operative pain. [27].

Many studies regarding ankyloglossia concentrate on the direct impact of frenulotomy on breastfeeding quality improvement. Ghaheri et al. [14] presented a statistically significant increase in milk transfer, improvement in the mother’s comfort, elimination of nipple pain as well as a remission of the newborn’s reflux symptoms after tongue-tie release. The procedure was equally effective in children with both anterior and posterior ankyloglossia. Pransky et al. [28] observed breastfeeding quality improvement after frenulotomy in 78% of newborns with anterior ankyloglossia and in 91% with posterior ankyloglossia. Wakhanrittee et al. [29] observed significant nipple pain reduction and increase of the LATCH index (Latch, *A*udible swallowing, nipple Type, Comfort, Hold) after frenulotomy. In the study by Muldoon et al. [30] as many as 91% of the mothers interviewed reported considerable decrease in nipple pain after the procedure, which led to significant improvement in breastfeeding comfort. Nipple trauma and pain during feeding is recognized as one of the main reasons behind premature weaning and switching to a bottle [30], thus it is such an essential aspect in the breastfeeding quality assessment in previously mentioned research. According to the Cochrane Database, a systematic review of randomized research published in 2017, in the short term, frenotomy reduces mothers’ nipple pain. However, authors have not found any unambiguous proof for longterm post-frenotomy improvement in breastfeeding. The review highlights methodological shortcomings of the majority of research, including small study groups, as well as lack of long-term observation and systematic follow-ups of infants from the control group who did not undergo the surgical procedure [3]. Nevertheless, one of the most recent studies presented the long-term efficacy of frenulotomy in improving breastfeeding rates and its influence on prolonged breastfeeding. Over the period of 3 months following the procedure, 80% of the mothers reported a complete resolution of nipple pain, 49% of the infants were exclusively breastfed, while 41% switched to mixed feeding (breastfeeding supplemented with some formula) [31]. Similarly, in the study by McGoldrick et al. [32] breastfeeding was continued for 3-4 months after the procedure in 76.8% of the newborns. Moreover, the literature emphasizes the importance of early surgical intervention after diagnosing breastfeeding disorders connected with ankyoglossia, because of the high risk of premature weaning. According to Donati-Bourne et al. [33] the majority of women give up breastfeeding and switch to the bottle if surgical procedure which brings some relief to the mothers is not performed in 4 weeks.

The literature increasingly emphasizes the necessity of multidisciplinary approach in the treatment of patients with ankyloglossia. Upon the diagnosis of a short lingual frenulum interfering in breastfeeding, the mother-infant dyad should first be referred to a lactation consultant. Nursing posture correction and myofunctional therapy, which stimulates proper sucking and rooting reflexes is recommended. Such stimulating exercises are also advised before and straight after any surgical intervention [34].

## Conclusions

Oral examination of a newborn infant in the first hours after birth should include thorough assessment of the lingual frenulum appearance and function. The recognition of problems in breastfeeding should raise the suspicion of ankyloglossia, which is listed as one of the possible causes of a changed sucking mechanism in infants. In order to systematize the examination, one of the suggested classifications may be applied, with particular emphasis on tongue function assessment, taking into account the possible occurrence of the less characteristic ankyloglossia posterior. Tongue-tie release (frenulotomy) should be performed quickly after diagnosing ankyloglossia that interferes in the sucking mechanism, in order to resolve breastfeeding problems as soon as possible. On the other hand, preventive frenulotomy in newborns with asymptomatic ankyloglossia is not recommended, due to the fact that the majority of infants with a short lingual frenulum do not experience problems in suction. Frenulotomy is a simple surgical procedure with little risk of complications, after which the newborn may immediately latch to the breast. The literature delivers a great number of studies confirming the efficacy of tongue-tie release in breastfeeding quality improvement with emphasis on decreasing mothers’ discomfort, nipple pain and trauma. Nevertheless, further research with improved methodology that focuses on long-term influence of surgical intervention on breastfeeding is still necessary.
